# Laparoscopic specimen extraction in vitro: preliminary experience

**DOI:** 10.1186/s12893-021-01300-5

**Published:** 2021-07-01

**Authors:** Yuanbi Huang, Tian Yi, Huajie He, Qiguang Li, Xian Long, Gaohua Hu, Qiwei Chen, Yongpeng Li, Rongchao Chen, Xianlin Yi

**Affiliations:** 1 Department of Urology, Cancer Hospital of Guangxi Medical University & Guangxi Cancer Research Institute, Nanning, 530021 People’s Republic of China; 2 College of Communication and Art Design, University of Shanghai for Science and Technology, Shanghai, 200093 China; 3 Department of Thyroid and Breast Surgery, Qichun People’s Hosiptal, Qichun, 435300 HuBei China; 4 Department of Urology, The People’s Hospital of Hezhou, Hezhou, 542800 Guangxi China

**Keywords:** Laparoscopic surgery, In vitro experiment, Specimen extraction, Ileostomy, Auxiliary incision, Urological neoplasms

## Abstract

**Background:**

The last procedure performed by the surgeon in laparoscopic surgery is to extract the specimen through the smallest incision possible. This experiment aimed to explore the maximum diameter of specimens that can be extracted through auxiliary incisions of different lengths and shapes by in vitro physical experiments.

**Materials and methods:**

We used the abdominal wall with the muscle layer, fixed on a square wooden frame, to simulate the human abdominal wall. Then, specimen extraction ports were made with circular, inverted Y-shaped and straight-line incisions of different sizes and lengths, and specimens of different sizes were made from tissues of different species. These specimens were extracted from different incisions with a force gauge. The tension value (N) was measured, and records were made of the length or diameter of the smallest auxiliary incision through which a given specimen could pass, as well as the largest specimen diameter that could pass through an incision of a given size. This experiment provides us with preliminary experience-based knowledge of how to choose the appropriate auxiliary incision for surgical specimen extraction according to the diameter of the specimen.

**Results:**

The maximum diameters of specimens that could be extracted with circular ostomy diameters of 2.4, 2.7 and 3.3 cm were 4.0, 4.5 and 6.0 cm, respectively. Specimens with diameters of 6.0, 8.0 and 10.0 cm could be extracted through inverted Y-shaped incisions with a length around the umbilicus of 1 cm and an extension length of 1.0, 3.0, and 4.0 cm, respectively. Moreover, these same specimens could be extracted through inverted Y-shaped incisions with a length around the umbilicus of 2 cm and extension lengths of 0.0, 1.0 and 2.0 cm. Tough tissue specimens (made from chicken gizzards) with diameters of 1.0, 2.0, 4.0 and 6.0 cm, respectively, could be removed through straight-line incisions measuring 1.0, 2.0, 3.0 and 4.0 cm in length.

**Conclusion:**

Along with preoperative imaging, surgical planning and trocar position, the shape and length of auxiliary incisions can be used to improve the extraction of specimens via laparoscopic surgery.

**Supplementary Information:**

The online version contains supplementary material available at 10.1186/s12893-021-01300-5.

## Background

Laparoscopy is commonly used in general surgery, urological surgery, obstetrics and gynecology. The retrieval of specimens after the operation is always a challenge for all surgeons. Small specimens can be removed through the trocar or trocar port, and some can be removed through the vagina [1] or anus [2, 3]

Moreover, in the field of gynecological surgery, it is feasible to perform mini-laparoscopy and single-site robotic surgery, where the surgical specimen can be removed at the end by in-bag transvaginal extraction instead of an external auxiliary abdominal incision. These techniques improve the cosmetic results in some gynecologic oncology surgery while ensuring complete specimen removal and good patient outcomes [4–7].

However, for intra-abdominal tumors, especially large specimens, surgical specimens are often removed through an auxiliary abdominal incision, it need to be removed through an auxiliary incision, and the surgeon must consider cosmetic appearance, minimization of trauma and complications, preservation of tumor integrity and the needs of the procedure itself. Some studies show that the minimal effect of laparoscopy is caused not by the length of the incision but rather by the reduction of both dehydration of the exposed abdominal organs and mechanical damage caused by gauze, glove contact and traction during surgery. In a prior study, no difference was observed in postoperative complications or postoperative recovery between auxiliary incisions measuring < 5 and > 5 cm in length [8]. However, a small auxiliary incision for specimen extraction can reduce postoperative pain and produce good cosmetic results. Therefore, the maximum diameter of specimens that can be extracted through auxiliary incisions of different lengths and shapes needs to be explored. To date, few studies have investigated the relationship between the size or shape of the specimen to be extracted and the length of the auxiliary incision. Casciola et al. [9] made a 3–5 cm incision around the umbilicus, starting at the trocar port in the superior part of the umbilicus, and successfully extracted specimens with a diameter of 6–7 cm without causing ischemic necrosis of the umbilicus. However, their study was limited to 3/4 of the way around the umbilicus, and the authors could not remove specimens larger than 7 cm. Some researchers [10, 11] have also removed nephrectomy specimens or uterine fibroids from the trocar port by morcellation, but the Food and Drug Administration (FDA) has issued a statement opposing the use of power morcellation for excision when the specimens are known or suspected to contain malignancy, as it increases the risk of disseminating malignant cells and worsening survival outcomes in patients with unexpected malignant neoplasms [12]. Fortunately, the specimen diameter can be measured by preoperative imaging, which enables the surgeon to design the shape and length of the auxiliary incision preoperatively based on the specimen size. In clinical practice, we have attempted to extract laparoscopic specimens (prostate and bladder) through an abdominal wall stoma in patients undergoing complete laparoscopic radical cystectomy and ileostomy, to extract laparoscopic radical prostatectomy specimens by enlarging the trocar port in the superior umbilicus into an inverted Y-shaped auxiliary incision, and to extract adrenal pheochromocytoma specimens by enlarging the trocar port into a straight-line auxiliary incision. However, no study has investigated the relationship between the size of the abdominal wall stoma or the length of the auxiliary incision and the diameter of specimens that can pass through the incision.

Based on the abovementioned theory and clinical practice, we used the porcine abdominal wall to simulate the human abdominal wall and extracted specimens in vitro to explore the relationship between the diameter of different specimens and the length and shape of auxiliary incisions.

## Materials and methods

### Experimental materials and group

**Fig. 1:**
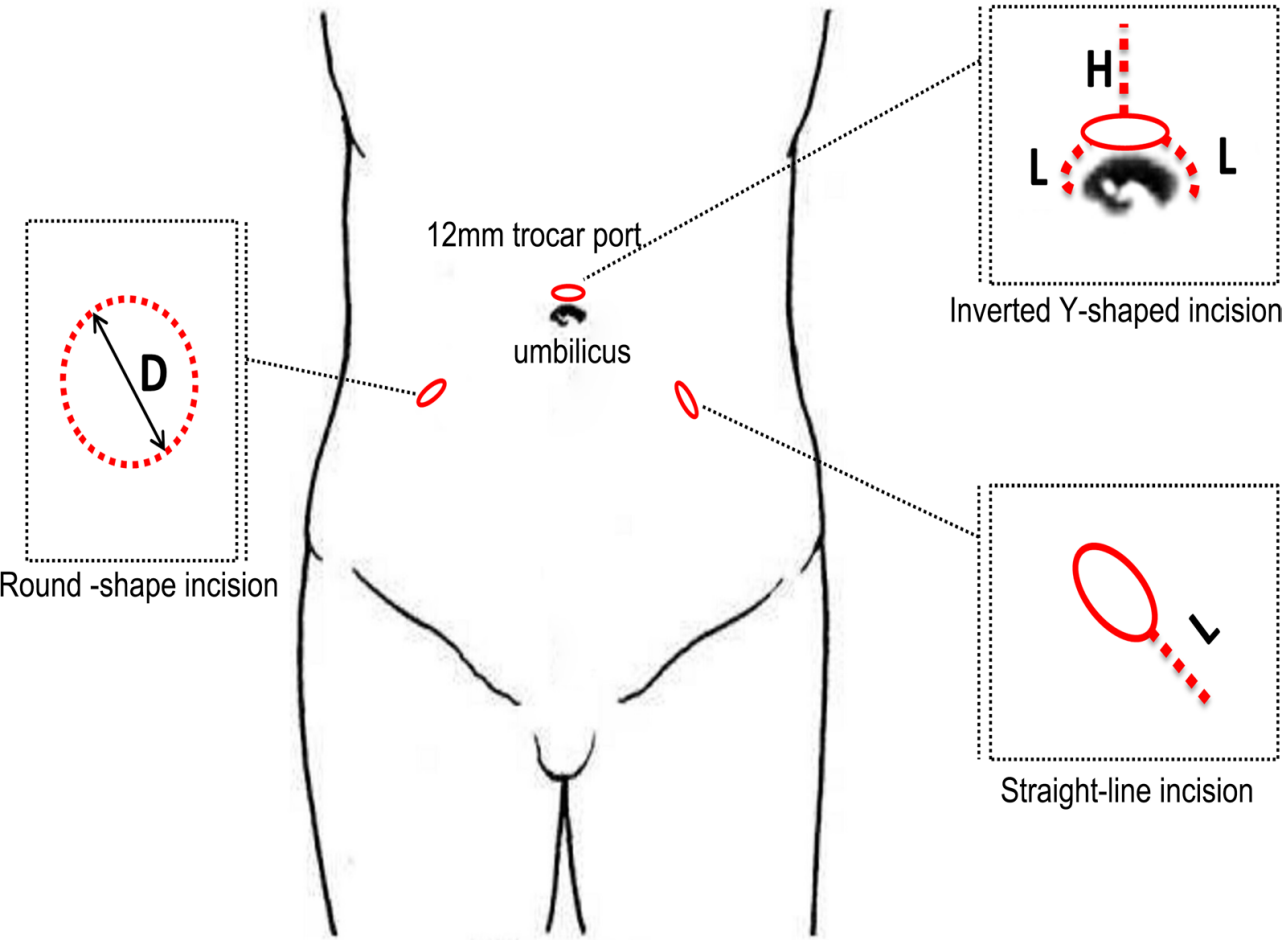
The three types of incisions used in experiment

**Fig. 2:**
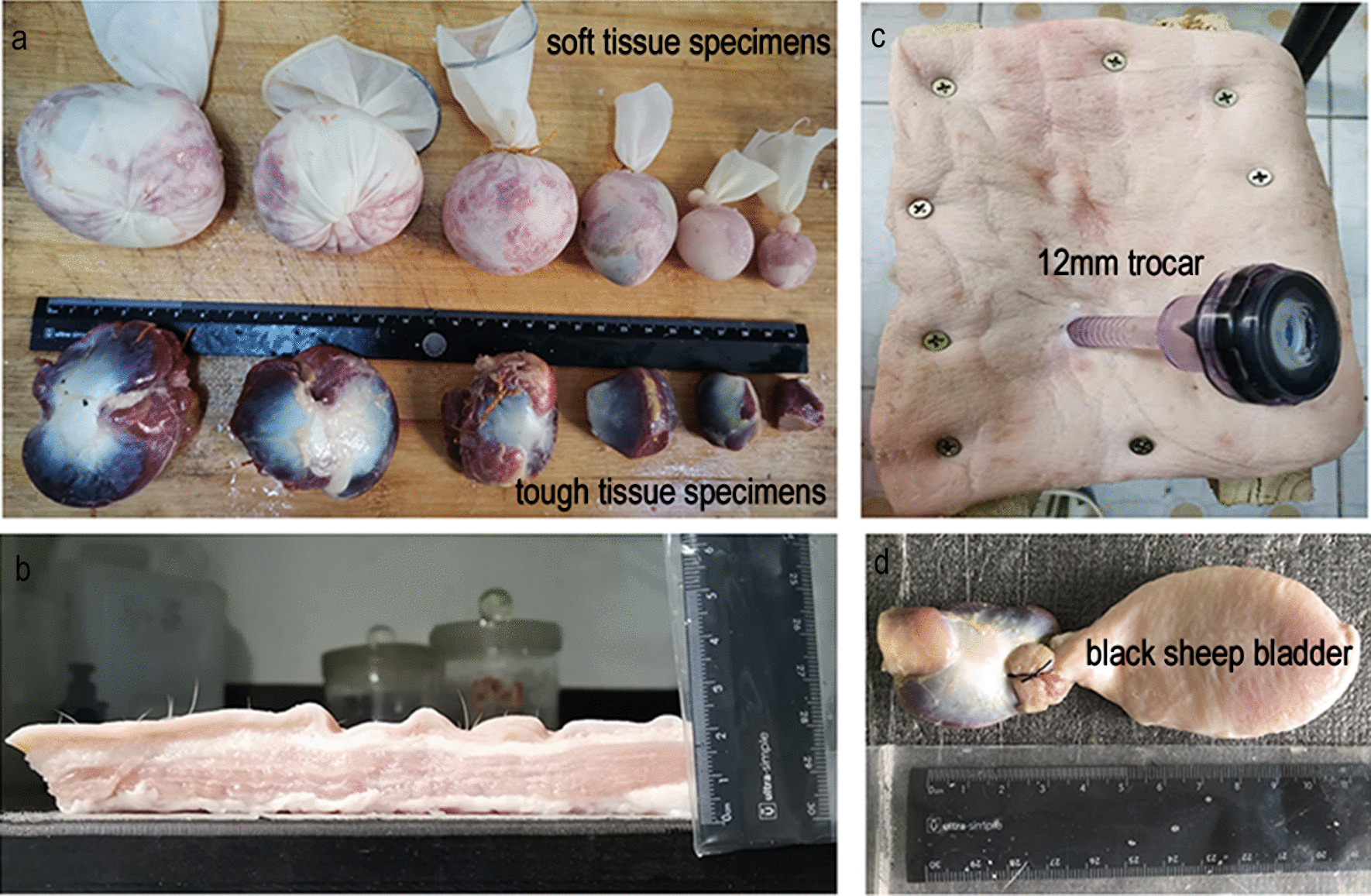
Images related to the experiment. **a** Specimens: tough tissue group (made from chicken gizzard) and soft tissue group (made from minced pork). **b** Abdominal model. **c** Pig's abdominal wall. **d** Prostate and bladder specimens (made from chicken gizzard and black sheep bladder)

Three pieces of porcine abdominal wall with a thickness of 2.0 cm were divided into three portions for repeated experiments. An in-house wooden frame was used to fix the porcine abdominal wall in place to simulate a human’s abdominal wall. Chicken gizzards, black sheep bladders and minced pig muscle were used to make specimens of different diameters (chicken gizzards were used for specimens with a diameter ≤ 6.0 cm, and the minced pork was packed in size 7.5 sterile surgical gloves to make specimens with a diameter ≥ 6.0 cm) (Fig. 2). The incision shapes were divided into three groups, namely, round, inverted-Y and straight-line shapes, each of which was made in a variety of sizes (Fig. 1). The round incisions were made in 5 graded sizes varying by intervals of 0.3 cm, with 3.0 cm as the central value (i.e., the sizes were 2.4, 2.7, 3.0, 3.3 and 3.6 cm). The inverted Y-shaped incisions were divided into two subgroups, according to the length of the incision around the umbilicus (L): L1 = 1.0 cm and L2 = 2.0 cm. Within each of these subgroups, five different values of extension length (H) were used: H1 = 0 cm, H2 = 1.0 cm, H3 = 2.0 cm, H4 = 3.0 cm, H5 = 4.0 cm. Straight-line incisions were made in 7 graded lengths: 1.0, 2.0, 3.0, 4.0, 5.0, 6.0 and 7.0 cm.

This study was approved by the Ethics Committee of the Affiliated Cancer Hospital of Guangxi Medical University (No. 20170308-4) and complied with the Declaration of Helsinki. Written informed consent was obtained from all participants. No live animals were involved in this experimental study, and all materials were purchased from supermarkets. The experiment was conducted without direct human involvement, and the data on human specimens in the manuscript are the result of measurements taken after the surgical specimens were removed in the usual way, not the result of the experimental design.

### Specimen preparation and experimental procedures

*Round group:* Chicken gizzards were made into circular specimens with diameters of 3.5, 4.0, 4.5, 5.0, 5.5 and 6.0 cm. They were sutured and connected to black sheep bladders. *Inverted-Y group:* The fingers of size 7.5 sterile surgical gloves were tied off tightly with knots, and the gloves were turned inside out to form pouches; the gloves were then packed with minced pork, shaped and tied tightly. The diameters of the specimens were set to 6.0, 8.0 and 10.0 cm. *Straight-line group:* The chicken gizzards and minced pork were made into two groups of specimens with different textures: a tough tissue group (made from chicken gizzards) and a soft tissue group (made from minced pork). Each group contained specimens with diameters of 1.0, 2.0, 3.0, 4.0, 5.0 and 6.0 cm (Fig. 2).

A porcine abdominal wall was fixed on a wooden frame with screws. The specimens were placed into a pouch, and the weight of each specimen was measured separately. A Shuangjie force gauge with an accuracy of 0.5 N and a total pulling force of 30 N was used for the measurements. Then, the specimens were extracted from each size of incision within each group of incision shapes. At the same time, the process of extracting each specimen was video recorded. Each experimental process was repeated three times, and the video was watched at the end of each experiment to read the three measurements of tensile force (N) (Additional file 1: Video S1).

### Observation index

The pulling force for each specimen extraction was recorded. If the pulling force was greater than the maximum reading of the force gauge but the device could still extract the specimen, the force was recorded as 30 N. If the specimen could be extracted manually but not with the force gauge, the force was recorded as 35 N. If the specimen could not be extracted by either method, the force was recorded as 0 N.

### Data processing and analysis

GraphPad Prism version 8.0 was used for mapping, and the results of the comparative analysis are presented as histograms. The relationship between the diameter of different specimens and the length and shape of the necessary auxiliary incision was analyzed.

## Results

### Round group

**Fig. 3:**
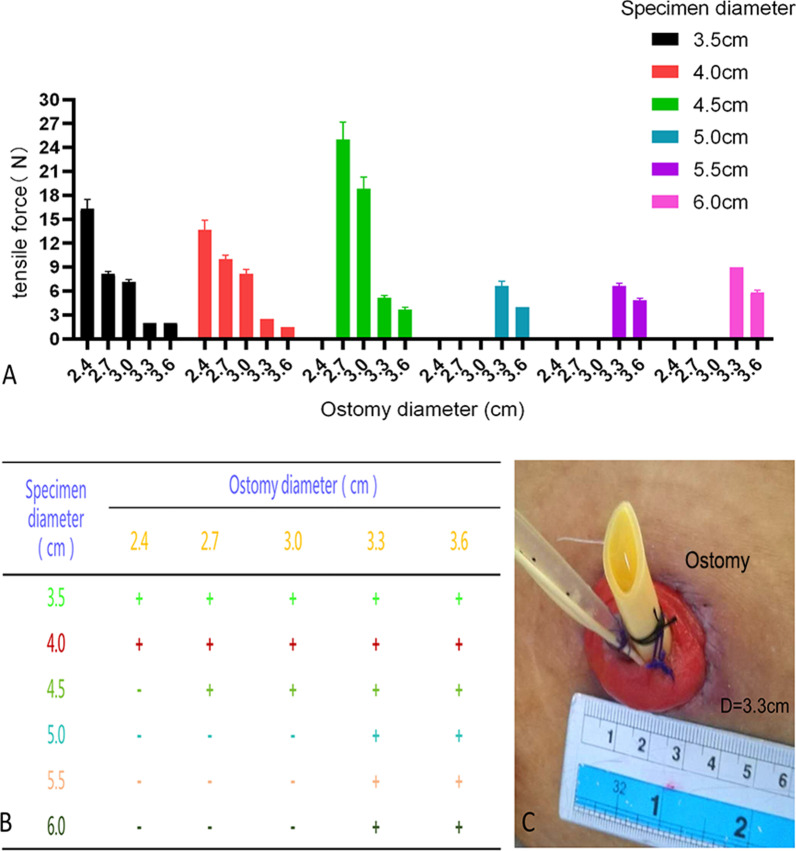
The result of the round-shaped group: **a** Histogram of the experimental results. **b** " + " for extractable, "−" for not extractable. **c** Stoma diameter size

As shown in Fig. 3, specimens with diameters of 4.0, 4.5 and 6.0 cm could be extracted from round stomata with diameters of 2.4, 2.7 and 3.3 cm, respectively.

### Inverted-Y group

**Fig. 4:**
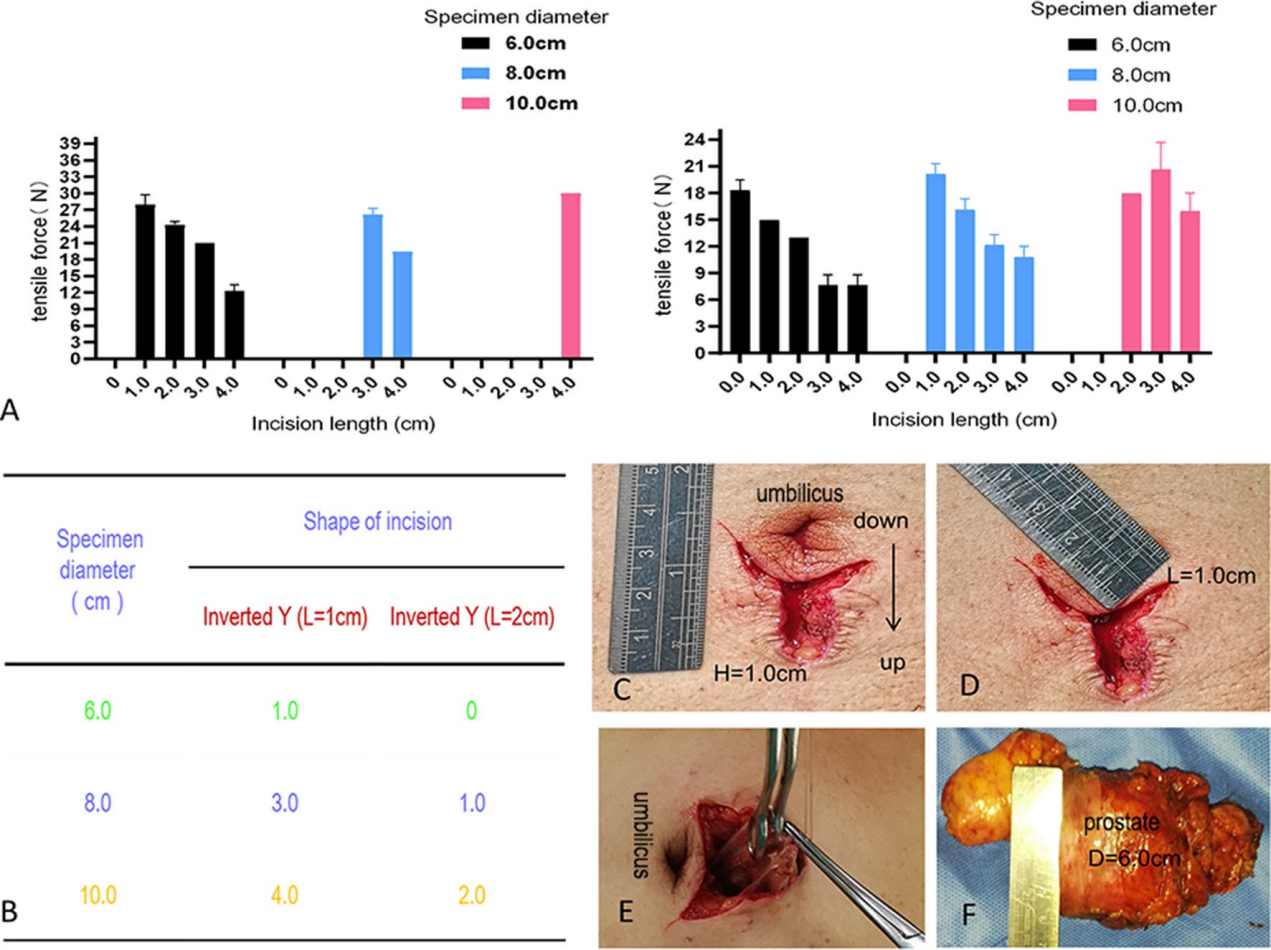
Results from the inverted-Y group. **a**, **b** Comparison of two types of incisions. **c** Extension length (H). **d** Length around umbilicus (L). **e** Deformation of incision during specimen extraction. **f** Specimen diameter

As shown in Fig. 4, specimens with diameters of 6.0, 8.0 and 10.0 cm could be extracted using inverted Y incisions with lengths around the umbilicus of 1 cm and extension lengths of 1.0, 3.0 and 4.0 cm; when the length around the umbilicus was 2 cm, extension lengths of 0.0, 1.0 and 2.0 cm, respectively, were needed to remove the specimens.

### Straight-line group

**Fig. 5:**
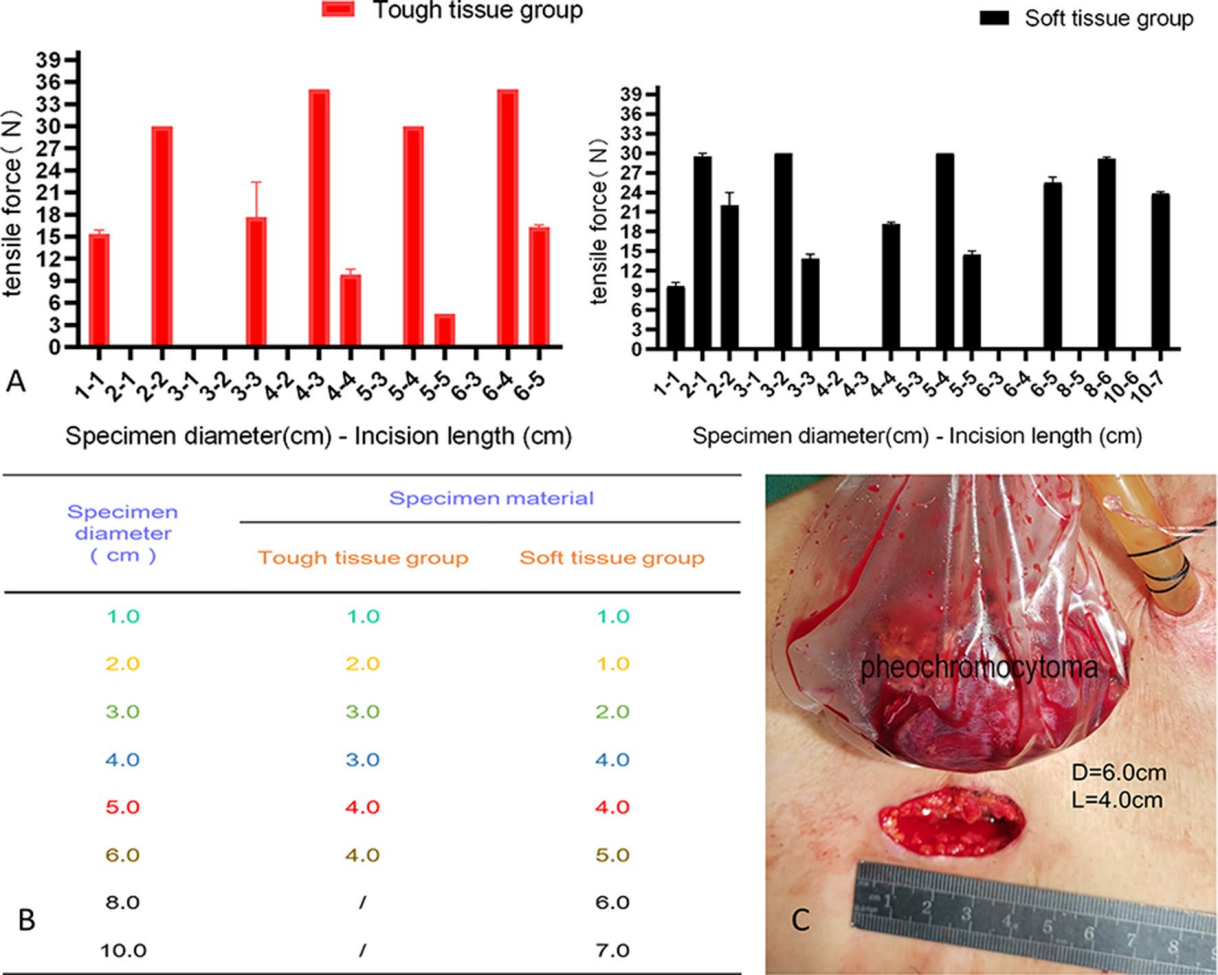
Results from the straight-line group. **a**, **b** Comparison of two types of incisions. **c** Specimen diameter and incision length

As shown in Fig. 5, the minimum incision lengths for the extraction of tough tissue specimens with diameters of 1.0, 2.0, 4.0 and 6.0 cm were 1.0, 2.0, 3.0 and 4.0 cm, respectively. Soft tissue specimens with diameters of 1.0, 3.0, 6.0, 8.0 and 10.0 cm could be extracted from incisions with minimum lengths of 1.0, 2.0, 5.0, 6.0 and 7.0 cm, respectively.

## Discussion

In laparoscopic surgery, smaller auxiliary abdominal incisions can offer both cosmetic advantages and important clinical implications, such as less postoperative pain, shorter hospital stays and a faster return to daily activities. However, no relevant research has indicated the relationship between the size of the abdominal wall stoma or the length of the incision and the specimen diameter. The method of extracting the specimen depends on the surgeon’s experience and personal preference. Moreover, regardless of the shape and size of the incision, several aspects should be considered: (1) keeping the wound small, (2) achieving good cosmetic results, (3) removing malignant tumor specimens intact, (4) taking urinary flow diversion into consideration and (5) minimizing complications of the auxiliary incision.

In this paper, we provide the first discussion of the relationship between the size of the abdominal wall stoma or incision and the specimen diameter by simulating postoperative specimen retrieval through in vitro physical experiments; our results are significant in that they can guide surgeons in choosing the appropriate shape and size for an incision based on the diameter of the specimen measured by preoperative imaging combined with the needs of the operation and the position of the trocar.

Ileostomy is widely used in the treatment of muscle-invasive bladder cancer in urological surgery. Surgical specimens in this procedure include the prostate and the bladder. The diameter of the ileum is approximately 3.0 cm [13], and the recommended diameter of the dermal stoma is 3.0 cm [14]. Thus, the diameter of the stoma should be 3.0 ± 0.3 cm. These findings, combined with the results of this experiment, indicate that an ileum abdominal wall stoma can be used to extract a specimen with a diameter of approximately 4.5–6.0 cm (Fig. 3). In clinical practice, we have attempted to extract laparoscopic specimens (prostate and bladder) from the abdominal wall stoma of patients undergoing complete laparoscopic radical cystectomy and ileostomy and verify that the extraction method is feasible.

In our experimental design, an incision in the shape of an inverted Y was mainly used to extract specimens from around the umbilicus. In clinical practice, many transabdominal laparoscopy trocar ports are located around the umbilicus, including the ports used for laparoscopic hepatectomy, splenectomy and resection of large abdominal tumors. The results of the experiment showed that specimens with diameters of 6.0, 8.0 and 10.0 cm could be extracted using inverted Y-shaped incisions with a length around the umbilicus of 1 cm and extension lengths of 1.0, 3.0 and 4.0 cm or with a length around the umbilicus of 2 cm and extension lengths of 0.0, 1.0 and 2.0 cm, respectively. Thus, this incision compensates for the shortcomings of the study by Casciola et al. to some extent [9]. We have also attempted to extract laparoscopic radical prostatectomy specimens in clinical practice by enlarging the trocar port in the superior umbilicus into an inverted Y shape, and the results are consistent with the present findings (Fig. 4).

Extending the trocar port as an auxiliary incision for specimen retrieval is the most common method of specimen extraction for most laparoscopic procedures. In tough tissue specimens (e.g., renal tumors, adrenal tumors and other tumors of the abdominal cavity), incisions with lengths of 1.0, 2.0, 3.0 and 4.0 cm could be used to extract specimens with maximum diameters of 1.0, 2.0, 4.0 and 6.0 cm, respectively. Moreover, for soft tissue specimens (e.g., spleen and liver tissue), incisions with lengths of 1.0, 2.0, 5.0, 6.0 and 7.0 cm could be used to extract specimens with maximum diameters of 1.0, 3.0, 6.0, 8.0 and 10.0 cm, respectively. We have also attempted to extract adrenal pheochromocytoma specimens by enlarging the trocar port into a straight line in clinical practice. The specimen diameter was approximately 6.0 cm, and the auxiliary incision was approximately 4.0 cm. This is consistent with the results of our experiment (Fig. 5).

**Fig. 6:**
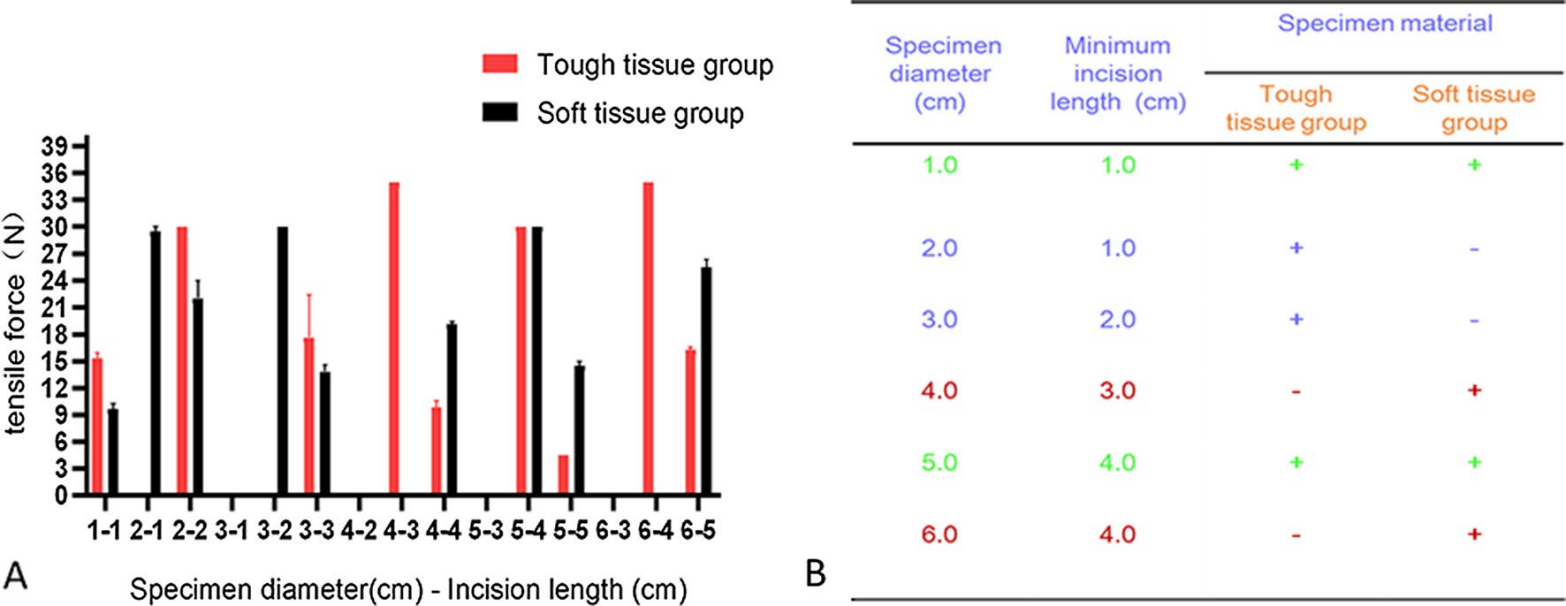
Comparison of the tough tissue group and the soft tissue group for the same incision length

**Fig. 7:**
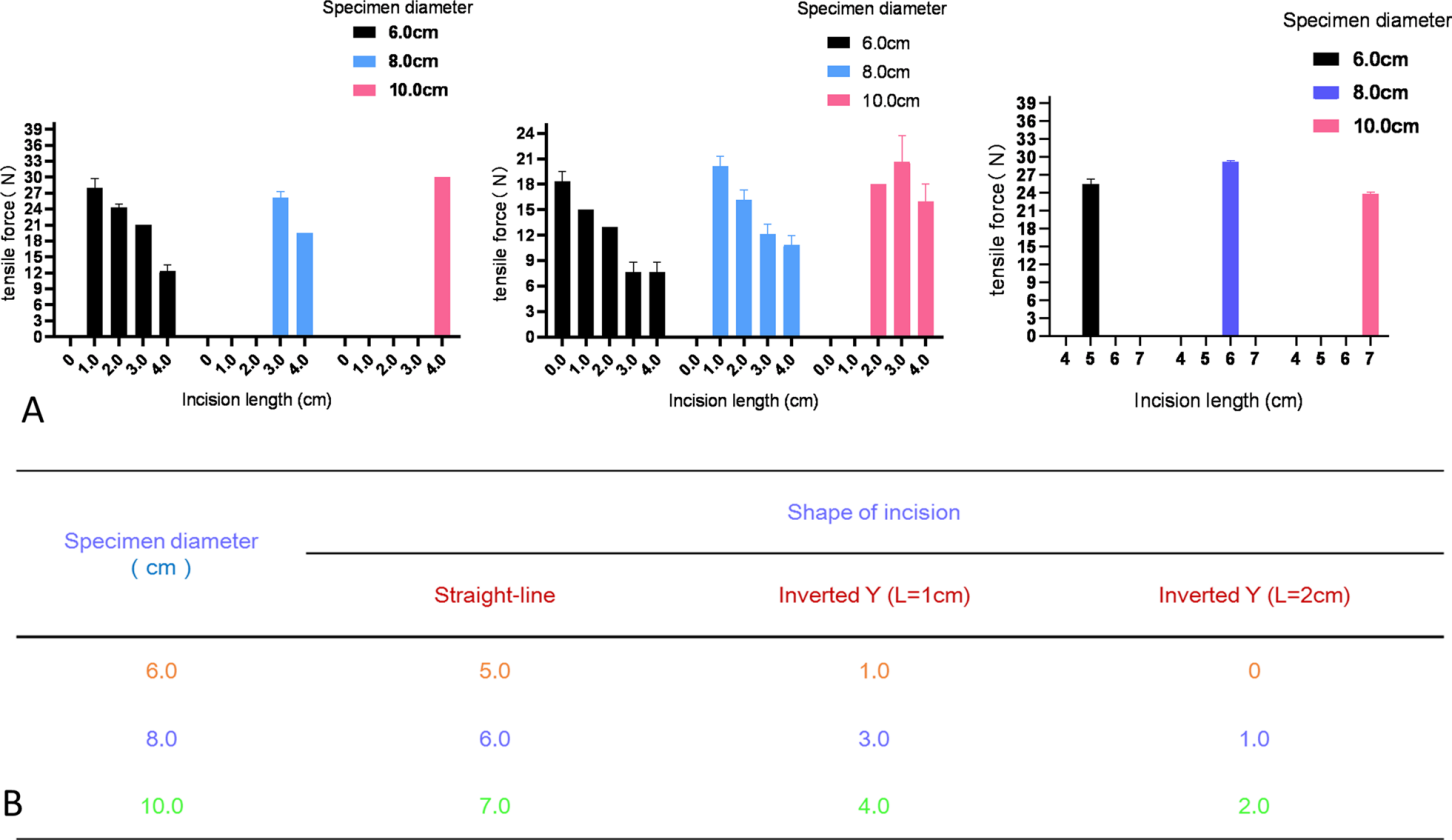
Comparison of straight-line and inverted-Y incisions for the same specimen diameter

In addition, when comparing specimens of the same diameter and different textures in the straight-line group, we found that soft tissue specimens with a diameter ≤ 3.0 cm were easier to extract than tough tissue specimens, whereas the opposite phenomenon was observed for specimens with a diameter > 3.0 cm. These findings may be related to the fact that soft tissues were squeezed and deformed more when they passed through the auxiliary incision, making it difficult to pull them through. In addition, when comparing straight-line incisions and inverted Y-shaped incisions for soft tissue specimens with diameters of 6.0, 8.0 and 10.0 cm, we found the inverted Y-shaped incision could reduce the length of the extended incision for large specimens, and the longer the incision was around the umbilicus, the more obvious this advantage was (Fig. 6, 7).

However, our experiment also has some shortcomings. First, we chose the muscular layer of the porcine abdominal wall instead of the whole abdominal wall. In the human body, the abdominal wall includes a peritoneal layer in addition to the muscular layer, which also increases the resistance to specimen extraction. Second, the specimens in this experiment had a limited variety of textures and shapes; differences in texture and shape between these specimens and real surgical specimens may result in differences between the experimental results and clinical practice. Third, to observe the measurement results, we extracted the specimen directly upwards with a force gauge (≤ 30 N). We manually removed the specimen only when the device was unable to extract the specimen (35 N). In clinical practice, the specimen needs to be extracted manually. Thus, our force measurements may differ from the amount of force that would be needed in clinical practice, which is the biggest limitation of our experiment.

In summary, although some studies have found that the auxiliary incision length is unrelated to complications, the fact that laparoscopic specimens can be removed through smaller auxiliary incisions not only improves the cosmetic results but also relieves postoperative pain, shortens the hospital stay and speeds the patient’s return to daily activities. Therefore, simulating the extraction process to determine the largest laparoscopic specimens that can be extracted through different sizes and shapes of auxiliary incisions is of great clinical significance.

## Supplementary Information

**Additional file 1: Video S1.** Video of experiment and partial operation.

## Data Availability

Additional unpublished data are available from the lead author on request.
